# A comparison of language control while switching within versus between languages in younger and older adults

**DOI:** 10.1038/s41598-023-43886-1

**Published:** 2023-10-05

**Authors:** Angela de Bruin, Heidi Kressel, Daisy Hemmings

**Affiliations:** 1 Department of Psychology, University of York, York, YO10 5DD UK; 2 Hull York Medical School, University of York, York, UK

**Keywords:** Human behaviour, Cognitive ageing

## Abstract

Word retrieval during language production slows down with age. However, bilinguals also require language control to manage language competition, in particular when switching languages to cues. The current study examined how this bilingual language control differs between younger and older adults. It also compared bilingual control, and age-group differences, to control that might be applied when alternating between responses within one language. In Experiment 1, 40 younger and 40 older monolingual adults completed a task alternating between noun and verb responses to pictures. The task showed costs associated with language control but these did not differ between age groups. Experiment 2 was completed by 50 older and 50 younger bilingual adults. Older adults showed larger switching costs than younger adults when switching between and within languages, suggesting they experienced more difficulty with reactive control. However, while older adults showed larger mixing costs than younger adults when using two languages in the dual-language environment relative to the single-language environment, they surprisingly showed smaller mixing costs than younger adults in the noun-verb within-language naming task. These findings show that language control, and the way it differs between older and younger adults, is not the same across within- and bilingual-language competition.

## Introduction

With ageing, several cognitive functions change, including language. Older adults are less accurate in confrontational naming tasks (cf.^1^), experience more tip-of-the-tongue moments where they cannot retrieve a specific word (e.g.,^2^), and are slower to name pictures (e.g.,^3^). Various mechanisms have been proposed to explain these changes in word retrieval with age. To some extent, slower language production can be explained through general slower processing in older adults (cf.^4^, but see^3^). However, focusing on language specifically, the Transmission Deficit Hypothesis^5^ argues that slower or poorer word retrieval is the consequence of weakened connections between different nodes in the language production system. In order to retrieve a word, lexical nodes for words related in meaning to relevant concepts become active. To produce those words, phonological nodes need to be activated too. With age, the transmission from lexical to phonological nodes in particular might weaken, leading to slower or insufficient activation for production.

While the majority of research has focused on changes in the retrieval of words, retrieval of target words alone might not be enough to allow for successful language production. We also need to manage competition from other, related words (e.g., “dog” when wanting to say “cat”). Older adults have greater difficulty suppressing irrelevant information while focusing on their goal^6^. This affects language too. For example, older adults are less coherent in story telling than younger adults, and this is modulated by their ability to suppress distracting relationships between words (e.g.,^7^).

This language control might be particularly important when people speak more than one language, as these bilinguals need to manage competition between their languages (e.g.,^8^). This control might be especially necessary when switching languages in response to different speakers, when bilinguals do not just need to select which words and language they want to use, but might also use inhibition to avoid interference from the previously used language^9^.

The current study examines how age influences the way people use language control during language production. Furthermore, it compares whether similar control mechanisms might be used when switching between words within a language versus when switching between languages. Experiment 1 firstly examined within-language switching in a noun-verb switching task in younger and older adults. In Experiment 2, we compared this within-language switching to between-language switching in bilinguals, to examine the involvement of language control processes in both, as well as potential age-related effects.

### Bilingual language control

Bilingual language control has frequently been studied through cued language-switching paradigms, in which participants name digits or simple pictures. A cue, such as a country flag, indicates which language has to be used for a given picture. These studies typically find that bilinguals are slower to name a picture when they have to switch languages than on non-switch trials when they can use the same language as on the previous trial (switching cost; e.g.,^10,11^). These cued switching costs reflect reactive control processes that bilinguals apply in order to respond to the language cue, activate the new target language, and avoid interference from the language used on the previous trial (cf.^12^ for a review).

In addition, these tasks can compare non-switch trials from dual-language contexts to single-language contexts in which bilinguals know they should just use one specific language. Typically, cued tasks show a mixing cost, with bilinguals needing more time in dual- than single-language contexts. This can reflect various mechanisms related to more proactive and attentional control (cf.^13^ for a review). A dual-language context requires closer cue monitoring to know which language to use. It also requires a bilingual to keep the language goal in mind and select the language accordingly. Finally, bilinguals might proactively balance the activation and/or inhibition of their languages to be able to flexibly use the two in dual-language contexts.

Prominent models of language control (e.g.,^9^) focus on language control in the form of inhibition, with bilinguals inhibiting the non-target language. How much inhibition is applied might depend on the language they are using. A more proficient language can create more interference and bilinguals are therefore argued to apply more control over the more proficient language (L1) than over less-proficient languages (L2;^9^). Although not always observed, unbalanced bilinguals who are more proficient in their L1 than L2 have been found to show an asymmetry in switching costs (e.g.,^10^), with larger costs when switching to the L1 than L2. The inhibitory control hypothesis^9^ explains this in terms of time needed to overcome previously applied inhibition. When using the L2, unbalanced bilinguals might strongly suppress the L1. When returning to the L1 on the next trial, time is needed to overcome the previously applied inhibition, leading to larger switching costs. Similar asymmetries have also been observed in terms of mixing costs (e.g.,^14,15^), with bilinguals responding faster in their L1 than L2 in single-language blocks but not in dual-language blocks. This shows language proficiency can also influence more proactive control, potentially with bilinguals over-inhibiting their L1 in dual-language contexts to allow for easier use of the L2 in the same context. In some cases, this can even lead to reversed dominance effects, with participants responding faster in their L2 than L1 in dual-language contexts (cf.^16^). However, these findings can also be explained without purely relying on inhibition. Instead (or in addition), bilinguals might over-activate their L2 to allow for easier use in a dual-language context^17^. Following these accounts, (relatively) faster L2 than L1 responses in a dual-language context might be the consequence of this L2 over-activation.

Studies assessing language changes with age in bilinguals have, similar to studies with monolinguals, shown slower or reduced lexical retrieval (e.g.,^18,19^). However, language control also appears to change with age. Several studies (e.g.,^20–22^) have found both switching and mixing costs to be larger for older than younger adults, suggesting both reactive and proactive control are affected. However, Hernandez and Kohnert^23^ only observed age-group differences in terms of mixing costs but not switching costs. In contrast, Weissberger and colleagues^21^ suggested that the age-group differences might be strongest for switching. Finally, Calabria et al.^24^ found no age-group differences on language switching beyond overall slower naming. Thus, based on the current literature, it remains unclear whether age affects both reactive and proactive language control.

### Language control in between- versus within-language competition

Furthermore, an open question is whether this language control is specific to resolving competition between languages or is also applied when resolving competition between words within a language. In particular when weaker relationships or words have to be retrieved, for example the less dominant meaning of an ambiguous word like bank^7^, top-down control might be applied. In bilingual language production models (e.g.,^9^), language control involves both bottom-up processes (e.g., an external cue telling the bilingual which language to use) and top-down control processes (e.g., inhibition and/or activation of specific languages). However, in influential models of (monolingual) word production, competition between words is often resolved just through bottom-up processes rather than top-down processes, for example by words being selected when they exceed the activation level of other words (e.g.,^25,26^). Inhibition can be part of this (e.g.,^27^), but typically in the form of lateral rather than top-down inhibition, so that word representations inhibit competitors directly. Top-down control is often not incorporated in these models (see^28^ for a discussion; and^29^ for an example of how top-down mechanisms can be implemented).

However, research has shown top-down control might be used when controlling responses in one language too. For instance, Piai and colleagues^30^ compared picture naming while ignoring distractors (e.g., picture of a dog while ignoring “cat”), colour naming while ignoring distractors in a Stroop task; and a non-linguistic task requiring object discrimination while ignoring its spatial position (Simon task). Incongruent trials in all conditions showed increased activation in the anterior cingulate cortex (ACC), an area associated with domain-general attentional control. Previous research has therefore also compared if markers associated with language control observed in bilingual language switching are also present in within-language switching. Markers such as mixing and switching costs and reversed dominance effects are difficult to explain without any form of top-down control (cf.^16,31^). Ivanova and Hernandez^31^ asked participants to name pictures using either basic-level responses (like “shoe”) or a subordinate name (like “sneaker”). Participants named pictures in blocks following the same rule or in blocks in which they had to alternate between the two rules. Switching costs, mixing costs, and asymmetries as well as reversed dominance effects were observed, leading the authors to conclude that, similar to bilingual language control, within-language competition might also trigger top-down control.

While within- and between-language control were not directly compared by Ivanova and Hernandez, Declerck and colleagues^32^ compared the two by asking French-English participants to switch languages or to switch between using formal or informal words in French. Both tasks showed significant switching costs, which correlated with each other and were comparable in size. This suggests similar control mechanisms were involved in the two types of switching. However, when manipulating the interval between the cue and stimulus, only language-switching but not register-switching costs were affected. Given that the cue-stimulus interval can be interpreted as active preparation of control processes in response to the cue, this suggests a difference in control mechanisms between language- and register-switching. Cattaneo and colleagues^33^ also observed some similarities and some differences when comparing between-language switching to a noun-verb within-language switching task. Switching costs did not correlate between the two tasks. In contrast, mixing costs did correlate between the tasks, although this appeared to be driven by the group of Parkinson’s Disease patients rather than the control group of older adults.

Finally, some studies have assessed within- versus cross-language interference by examining language switching in a paradigm with congruent or incongruent Stroop trials. Stroop trials require participants to name the physical colour of a word, which is either the same as the word itself (e.g., “red” in red ink, congruent) or different (e.g., “green” in red ink, incongruent). Some studies have shown larger language-switching costs in incongruent Stroop contexts (e.g.,^34,35^), suggesting that managing Stroop interference and between-language interference might recruit the same cognitive resources. However, not all studies have replicated these findings (e.g.,^36^).

## Experiment 1

### Introduction

Thus, although there are suggestions in the literature that within-language switching might use language control too, it remains unclear whether these control processes are specific to within- or between-language competition or whether they are more general and comparable across the two. In the first Experiment, we tested a within-language switching task, with the aim of comparing this task to between-language switching in Experiment 2. We chose a task (similar to^33^) asking participants to name pictures while either using a noun to describe the object or a verb describing the action associated with it (e.g., “book” or “read”). The advantage of using this paradigm is that previous research has shown nouns are processed faster than verbs (e.g.,^33,37,38^, cf.^39^ for a review including lesion research), allowing us to identify a priori the easy-to-use rule (using nouns, similar to the L1 being the easier to retrieve language) and the more difficult rule (using verbs, similar to words in the L2 being more difficult to retrieve).

We furthermore examined how different types of control, if used within one language, might change with age. Previous research on bilingual control (as also further assessed in Experiment 2) has suggested that age can influence both reactive and proactive control processes. However, research on non-linguistic control has suggested age might affect proactive control more than reactive control. Several meta-analyses (e.g.,^40–42^) have shown age effects predominantly occurring on tasks requiring participants to mix or use multiple tasks. For instance, Wasylyshyn and colleagues^42^ showed clear age-group differences on task-mixing costs, suggesting older adults have increased difficulty completing two tasks in parallel, potentially also in relation to changes in working memory coordinating and maintaining multiple task rules within one context. However, the same meta-analysis showed no age-group differences in terms of task-switching costs, suggesting that more reactive control mechanisms are influenced less by age.

This leaves open the possibility that age also influences proactive control more strongly in language contexts. However, language control and executive control used in non-linguistic tasks might only overlap partially (e.g.,^43,44^). Furthermore, two studies comparing age effects on bilingual switching and task switching showed different patterns depending on the task. Calabria et al.^24^ only observed age-group differences on non-linguistic switching costs but not on linguistic switching costs. Weissberger et al.^21^ showed clear age-group differences in both tasks but while the language-switching task showed differences in terms of both switching and mixing costs, the non-linguistic switching task only showed age-group differences on the mixing cost errors (in line with e.g.,^42^).

#### Current study

Experiment 1 thus had two aims. First, it examined if monolinguals show effects typically associated with language control while switching between different types of words within one language. Specifically, we asked participants to name pictures while either using the noun to describe the object (e.g., “bed”) or the verb to describe an action associated with the object (e.g., “sleep”). They completed this task in single-rule contexts (always using the noun or verb) and dual-rule contexts, where they had to alternate between nouns and verbs in response to a cue. We examined whether participants showed switching costs (often used as a reflection of reactive control) and mixing costs (often used as a reflection of proactive control and effort associated with coordinating and maintaining multiple rules in mind). Furthermore, we examined asymmetries in these costs (with easier to retrieve responses being affected more by mixing or switching), as these in particular are often interpreted in line with control and inhibition (e.g.,^16^).

As a second aim, Experiment 1 was completed by younger and older adults. If older adults have more difficulty with proactive control, we expected larger mixing costs in that age group, while their switching costs should be larger if their reactive control is affected. Finally, to examine whether any age-related differences are specific to language control, we also compared older and younger adults on a non-linguistic switching task in which they had to sort digits according to size (smaller or larger than 5) or parity (odd/even). For the non-linguistic switching task, we expected (in line with meta-analyses such as^42^) proactive control (mixing costs) to differ between age group while reactive control (switching costs) might be not or less affected.

### Methods

#### Participants

Experiment 1 was completed by forty older adults (*M*age = 68.65 years old, *SD* = 3.82, range = 65–77) and forty younger adults (*M*age = 22.28, *SD* = 3.49, range = 18–29). Participants provided informed consent at the start of the study. Ethics approval was given by the ethics committee in the Department of Psychology, University of York. The study was conducted in accordance with the Declaration of Helsinki, with the exception of the requirement for pre-registration. All participants reported that they were native speakers of English, monolingual, and had not been diagnosed with language or reading difficulties, a neurological disorder, or cognitive impairment. They also reported having normal or corrected-to-normal vision and hearing and were not taking medication that could influence their concentration. Given that the study was conducted online, we did not use an assessment of cognitive functioning (e.g., MMSE). However, we used Prolific’s screening criteria to only invite participants without a history of cognitive impairment or dementia, which participants also confirmed in the questionnaire. Sample size was determined based on effect sizes typically observed for switching and mixing effects in bilingual switching tasks, which can be observed with >95% power with this sample size. Given that age-group differences have not been studied on noun-verb switching tasks, we could not run a power analysis for the interaction between age and language control. Therefore, we followed the recommendation^45^ to use 40 participants and 40 trials per combination of conditions for sufficient power in mixed-effect analyses.

Within the group of younger adults, 15 identified as male and 25 as female. Within the group of older adults, 22 identified as male and 18 as female. In terms of highest level of education achieved, 16 younger adults and 23 older adults reported having completed a graduate degree. An additional 12 participants were tested but excluded because they showed low accuracy (below 70% on the noun-verb switching task; N = 6), reported being bilingual (N = 5), or reported having a neurological disorder or cognitive impairment (N = 1).

#### Design

Participants completed two switching tasks. The linguistic noun-verb switching task asked participants to name pictures using nouns (e.g., “bed”) and verb (e.g., “sleep”) responses. The non-linguistic switching task asked participants to respond to digits based on either their size or parity. Both tasks included a single-rule block (only requiring participants to follow one rule, for example always using nouns) and a dual-rule block, in which participants alternated between rules. Within the dual-rule block, some trials were switches (different rule than on previous trial) while other trials were non-switches (same rule as on previous trial).

Per task, we conducted one set of analyses focusing on mixing effects (difference between single-rule and non-switch dual-rule trials) and one focusing on switching effects (difference between switch and non-switch trials within the dual-rule block). Each analysis included trial type (switch/non-switch; or non-switch/single-rule), rule (noun/verb for the linguistic task and size/parity for the non-linguistic task), and age group as predictors, and accuracy and reaction times as the dependent variables.

#### Materials

For the linguistic noun-verb switching task, we selected twenty pictures from the MultiPic database^46^. We selected pictures that showed high naming agreement for the nouns. Furthermore, we ensured each picture had a concrete action associated with it that could be used when responding to the “verb” rule. Feedback was gathered from three speakers to choose pictures that only had one likely action associated with it. For instance, the picture of a chair has the specific action “sit” closely linked to it. An overview of the stimuli is provided on the OSF page: https://osf.io/rdfcs/. Verb and noun picture names were matched in terms of frequency and number of phonemes (see OSF page). All verbs were one syllable long and participants were asked to just name the infinitive, without “to”. All nouns were one or two syllables long. In the non-linguistic switching task, participants saw a digit between 1 and 4 or between 6 and 9 on the screen and responded to its size or parity with a button press.

Within each task, we used two written cues per rule to avoid a confound between cue and rule switching^47^. This way, even when the rule stayed the same (e.g., two trials in a row requiring a noun response), the cue still changed. In the linguistic noun-verb task, the words “NOUN” or “OBJECT” and “VERB” or “ACTION” were used as cues. In the non-linguistic task, the words “MAGNITUDE” or “SIZE” and “PARITY” or “EVENNESS” were used.

#### Procedure

Participants were recruited through Prolific.co and completed the study on Gorilla.sc^48^. After reading the information sheet and providing consent, participants first completed a short questionnaire. This questionnaire asked them about their age, gender, and education. Additional questions were included to ensure participants met our eligibility requirements (see “Participants” section). Afterwards, participants completed the linguistic and non-linguistic switching tasks, with the order counterbalanced across participants.

The linguistic noun-verb task started with a microphone check, in which participants were asked to record their verbal response to one picture. They could play back their response and were asked to only continue if they could hear themselves clearly in the recording. Next, they saw the picture familiarisation phase, in which they saw each picture with the written noun and verb responses next to it. This was included so that participants knew which pictures to expect and did not have to think about what objects and actions they were seeing while naming them.

The main task included two single-rule blocks (one for nouns and one for verbs, with the order counterbalanced across participants), the dual-rule block, and another two single-rule blocks. The first two single-rule blocks were preceded by two practice trials with pictures that were not part of the main experiment; the dual-rule block was preceded by four practice trials. Each picture was repeated once within each single-rule block, leading to a total of 80 single-rule trials (40 per rule). The dual-rule part included 160 trials, which were evenly distributed across trial type (switch/non-switch) and rule. Each picture was presented eight times. Stimulus order was pseudo-randomised so that pictures did not appear twice in a row and there were no more than four trials of the same rule or trial type in a row. Each picture was preceded by a fixation cross for 500ms and stayed on the screen for 3500ms, regardless of when the response was given.

The non-linguistic task followed the same set-up. Rather than giving verbal responses, participants saw four buttons on the screen: “<5”, “>5”, “even”, “odd”. Participants were shown an example and asked to click on “<5” or “>5” when following the magnitude rule and on “even” or “odd” when following the parity rule. The next trial was started as soon as a participant gave a response or moved on after ten seconds if no response was given. Before the fixation cross was shown, participants were asked to click on a button in the middle of the screen to reset the mouse position before the start of each trial.

#### Data analysis

The data and analysis code can be found here: https://github.com/AMTdeBruin/Bilingual-switching-ageing. They are also available at https://osf.io/rdfcs/. Verbal responses in the linguistic noun-verb switching task were recorded and scored for accuracy and naming onset time. A response was scored as correct if it was the target word or a similar alternative (e.g., “gift” instead of “present”). Responses were scored as incorrect if there was no response, if a different word was used (e.g., “apple” instead of “strawberry”), or if a response was given in the wrong language or combining two languages. Reaction times (RTs) relative to onset of picture presentation were determined using CheckVocal (cf.^49^, using CheckFile).

We analysed age-group differences for each task separately, with one analysis focusing on switching costs (switch and non-switch trials only) and one analysis on mixing costs (non-switch and single-rule trials). Accuracy was not at ceiling and analysed using generalized linear mixed-effects models. RTs were analysed through linear mixed-effects models, using package *lme4* (version 1.1.33) and *lmerTEST* (version 3.1.3) in R (version 4.3.0).

All analyses included Rule (noun coded as $-0.5$; verb as 0.5; or size coded as $-0.5$ and parity as 0.5); Age group (younger adults coded as $-0.5$; older adults as 0.5); and trial type (switching: non-switch coded as $-0.5$ and switch trials as 0.5; mixing: single-rule coded as $-0.5$ and non-switch trials as 0.5). For the accuracy analysis, we excluded trials in the dual-rule condition preceded by a break as they did not have a trial type (switch or non-switch). For RT analyses, we excluded incorrect responses and trials preceded by a break, no response, or wrong-language response. Prior to the RT analyses, we also removed RT outliers that fell 2.5*SD* above or below the mean by participant and condition (1.6% of correct noun-verb task trials and 1.9% of non-linguistic task trials,^50^). Visual inspection of the RT data showed that they were not normally distributed and we therefore conducted the analysis with log-transformed RTs. Descriptive statistics provided in the text and tables or figures are based on untransformed data.

All analyses started with maximal models including by-participant and by-item intercepts and all within-participant/-item slopes. When models did not converge, we first removed correlations between slopes and intercepts, followed by removal of item slopes that explained the lowest amount of variance. Details about the random-effects structure of the converging models are provided per results table.

In the non-linguistic switching task, three participants performed below 70% correct. We did not exclude these participants as they performed above the threshold for the main task of interest, the linguistic noun-verb switching task. However, we reran the non-linguistic analyses without these three participants too, which showed the same results as when the analyses included them.

Finally, in addition to these analyses, we also examined whether the mixing and switching costs were related across the linguistic and non-linguistic switching tasks. For each participant, we therefore computed their switching cost (RT difference between switch and non-switch trials) and mixing cost (RT difference between non-switch and single-rule trials) per task and conducted correlational analyses comparing the costs across tasks.

### Results

#### Linguistic noun-verb switching task

##### Switching cost

***Accuracy***

Mean accuracy scores by age group, trial type, and rule type are presented in Supplementary Table S1. Overall, accuracy was high, with means over 90% in all conditions. When errors were made, they mostly concerned responses following the incorrect rule (e.g., noun rather than verb). The analysis (see Supplementary Table S2) revealed a main effect of trial type, reflecting that participants made more errors on switch than non-switch trials (see Supplementary Table S1). This error switching cost did not interact with age group or rule.

***Reaction times***

Participants’ mean RTs per condition can be found in Table 1. Table 2 shows the full results of the switching-effect analysis. Participants showed a significant switching cost, with slower responses on switch trials (*M* = 1416, *SD* = 204) than on non-switch trials (*M* = 1364, *SD* = 208). While older adults (*M* = 1418, *SD* = 196) responded more slowly numerically than the younger adults (*M* = 1361, *SD* = 209), this difference did not reach significance in the dual-rule condition only. Of main interest for the current study, the switching cost did not differ between younger and older adults (see Table 1). None of the other main effects or interactions were significant either.

**Table 1:** Mean RTs (and standard deviations) in the noun-verb switching task in Experiment 1.

	Younger adults	Older adults
Single-rule		
Noun	1032 (171)	1098 (160)
Verb	1078 (190)	1179 (184)
Non-switch		
Noun	1350 (211)	1420 (200)
Verb	1308 (219)	1384 (221)
Switch		
Noun	1413 (238)	1469 (199)
Verb	1373 (202)	1406 (205)
Mixing cost		
Noun	319 (136)	321 (176)
Verb	230 (155)	205 (122)
Switching cost		
Noun	63 (109)	49 (93)
Verb	65 (81)	22 (93)

**Table 2:** Outcome of the linear mixed effect models for the linguistic noun-verb switching task in Experiment 1.

Fixed effects	Estimate	Standard error	t-value	*p* value
Switching analysis				
Intercept	7.206	0.019	383.598	< 0.001
Switching	0.043	0.008	5.191	< 0.001
Rule	− 0.032	0.019	− 1.702	0.103
Age group	0.051	0.032	1.579	0.118
Switching × Age group	− 0.014	0.01	− 1.462	0.148
Switching × Rule	0.002	0.019	0.084	0.934
Age group × Rule	− 0.012	0.015	− 0.786	0.435
Switching × Age group × Rule	− 0.01	0.021	− 0.481	0.635
Mixing analysis				
Intercept	7.074	0.018	388.526	< 0.001
Mixing	0.22	0.012	17.754	< 0.001
Rule	0.011	0.018	0.6	0.554
Age group	0.071	0.031	2.312	0.023
Mixing × Age group	− 0.024	0.021	− 1.112	0.27
Mixing × Rule	− 0.086	0.015	− 5.781	< 0.001
Age group × Rule	0.008	0.017	0.444	0.659
Mixing × Age group × Rule	− 0.025	0.024	− 1.041	0.305

The final model for the switching analysis included by-subject and by-item random intercepts, as well as all by-subject random slopes and all by-item slopes apart from age × switching. The final mixing model included all intercepts and slopes, after removal of correlations.

##### Mixing cost

***Accuracy***

The full results of the mixing cost error analysis are provided in Supplementary Table  S2. The error analysis revealed a mixing cost, with participants making fewer errors in the single-rule condition than in the dual-rule condition (see Supplementary Table  S1). No further effects of, or interactions with, age group or rule were found.

***Reaction times***

The full results of the mixing cost RT analysis can be found in Table 2, with descriptives shown in Table 1. Participants showed a significant mixing cost, with slower responses on non-switch trials (*M* = 1364, *SD* = 208) than on single-rule trials (*M* = 1096, *SD* = 170). Across the single-rule and non-switch trials, older adults (*M* = 1265, *SD* = 166) also responded more slowly than younger adults (*M* = 1186, *SD* = 177). Of main interest for the current study, the mixing cost did not differ significantly between age groups (see Table 1).

The rule (noun or verb responses) influenced the mixing cost, such that the mixing cost was larger for noun decisions (*M*mixing cost = 320, *SD* = 156) than for verb decisions (*M*mixing cost = 217, *SD* = 140). While noun responses were faster than verb responses in the single-rule condition, the opposite was observed in the dual-rule condition (see Table 1).

#### Non-linguistic task

##### Switching and mixing costs

***Accuracy***

Supplementary Table  S3 presents the accuracy by condition in the non-linguistic task, with Supplementary Table  S4 providing the results from the analyses. The error analysis showed a significant switching effect, but this was in the opposite direction of what was expected: accuracy was higher on switch trials than on non-switch trials (see Supplementary Table  S3). This was especially the case for the size-rule trials. There was also a significant mixing cost, with better performance in the single-rule condition. This interacted with age, but contrary to the prediction, this mixing cost was larger for younger than older adults (see Supplementary Table  S3).

***Reaction times***

Supplementary Table  S5 provides the full results from the switching- and mixing-effect analyses. Both analyses (see Table 3) showed that older adults responded more slowly (*M* across all trial types = 1743, *SD* = 298) than younger adults (*M* across all trial types = 1264, *SD* = 281). Surprisingly, no switching effect was found (see Table 3), reflecting that switch (*M* = 1645, *SD* = 405) and non-switch RTs (*M* = 1687, *SD* = 437) were not significantly different. If anything, people were responding more slowly to non-switch trials, contrary to the hypothesis. There was a mixing effect, reflecting that responses were fastest on single-rule trials (*M* = 1220, *SD* = 354).

The switching effect was not significantly different between age groups. The mixing cost did differ between younger and older adults. In terms of untransformed RTs, older adults showed slightly higher mixing costs (see Table 3). However, in terms of log RTs (as used in the analysis), older adults’ mixing cost was smaller (*M* cost = 0.29, *SD* = 0.12) than that of younger adults (*M* cost = 0.38, *SD* = 0.13). Given that the direction differed between log RTs and untransformed RTs, and considering the large difference in overall RTs between age groups, we conducted an additional analysis using z-scored RTs. The interaction between age and mixing costs was not significant in this analysis ($\beta$ = $-$ 0.062, *SE* = 0.072, *t* = $-$ 0.861, *p* = 0.392).

**Table 3:** Mean RTs (and standard deviations) in the non-linguistic switching task in Experiment 1.

	Younger adults	Older adults
Single-rule		
Size	1020 (305)	1464 (312)
Parity	962 (216)	1433 (284)
Non-switch		
Size	1464 (436)	1934 (389)
Parity	1434 (396)	1925 (352)
Switch		
Size	1415 (328)	1873 (367)
Parity	1374 (247)	1920 (369)
Mixing cost		
Size	444 (257)	470 (261)
Parity	472 (295)	492 (235)
Switching cost		
Size	− 49 (184)	− 61 (194)
Parity	− 59 (237)	− 5 (178)

#### Correlations

Finally, we examined whether the linguistic and non-linguistic switching and mixing effects were correlated. Non-linguistic and linguistic noun-verb switching costs were not significantly related: *r*(78) = $-$ 0.118, *p* = 0.298. Mixing costs showed a small correlation across tasks, although this was not significant with the Bonferroni corrected *p* value of 0.025: *r*(78) = 0.241, *p* = 0.031.

### Discussion

Experiment 1 tested a noun-verb switching task to examine whether markers of language control (switching and mixing costs) frequently observed in bilinguals are observed when switching within a language too. It furthermore compared those effects between younger and older adults, and compared the costs to a non-linguistic switching task. The noun-verb switching task showed both switching and mixing costs, but no age-group differences. The non-linguistic switching task showed, surprisingly, only a mixing and no switching cost.

#### Within-language noun-verb switching

The noun-verb switching task showed that participants needed more time when switching between rules (switching cost) as well as when using two rules in a dual-rule condition compared to a single-rule condition (mixing cost), in line with^33^. The presence of mixing costs suggests that participants recruited additional resources to monitor cues and to select responses according to the rules indicated by the cues. The presence of switching costs furthermore shows that participants experienced competition between the different responses. Interestingly, the noun-verb mixing cost also showed an asymmetry, with participants showing larger RT mixing costs for the easier rule (object/noun naming). Although this did not reach significance, a similar numerical pattern was present in the accuracy analysis too, showing that the RT asymmetry was not the consequence of a speed-accuracy trade-off for, for example, the noun responses.

This pattern is in the same direction as asymmetries often observed in the bilingual production literature, either regarding switching costs (e.g.,^10^) or mixing costs (e.g.,^15^). These asymmetries are often explained through a form of language control, with people potentially inhibiting the easier responses to allow for flexible use of responses that are more difficult to retrieve. The finding that this asymmetry concerned mixing costs suggests that participants might have proactively inhibited noun responses to more easily use verb responses too. Alternatively, or additionally, they might have proactively over-activated the verb responses (e.g.,^17^). This suggests some form of control is involved even in noun-verb switching. If the costs observed were purely due to more difficult words requiring more time to reach their activation threshold, the verbs should have shown larger mixing or switching costs. Instead, monolingual production might use language control too, in particular when speakers are required to alternate between responses in high-competition situations such as our dual-rule task.

Interestingly, age did not influence the mixing nor switching costs. More generally, within the dual-rule context, no overall age-group difference in terms of overall naming RTs was observed either. This is surprising as overall slower naming in older adults would be expected to be present, or even most pronounced, in the most-difficult dual language context. By comparing this task with the bilingual switching task in Experiment 2, we aim to better understand whether these groups of older adults performed at a similar level as younger adults regardless of the task or whether an absence of age-group differences is specific to within-language switching.

#### Task switching

Experiment 1 also included a (non-linguistic) task switching paradigm. Surprisingly, this task did not show a switching cost in terms of RTs and showed a switching benefit in terms of accuracy, contrary to a vast literature observing costs when switching between a range of tasks^51^. A large mixing cost was observed, showing that using different rules was more difficult than being in a single-rule condition. It is possible that the dual-rule condition was very difficult overall, even when not having to switch between rules. Even on non-switch trials, participants had to choose between one of two possible responses corresponding to the rule (for example, smaller or larger than 5 within the size-rule task). Responses in a linguistic noun-verb naming task might be more automatic, as language production is natural and people are fast at retrieving a word like “horse” in response to a picture. In contrast, determining whether 7 is smaller or larger than 5 might be difficult even on non-switch trials and is not a task people frequently perform in daily life. Furthermore, this might have been even more difficult in the online set-up, where participants had to click on, and remember, buttons corresponding to these options. Indeed, both age groups responded relatively slowly while doing this task. Older adults were significantly slower than younger adults. However, this larger age-group difference in terms of overall RTs could be explained by the general difficulty of the task and the absence of a strict time-out.

The very large mixing cost further confirms that the dual-rule context was difficult for both age groups and that participants might have put in much additional effort to respond to both non-switch and switch trials. A small difference in mixing costs between age groups was observed in the analysis on log RTs, although the direction varied for untransformed and log-transformed RTs. While the untransformed RTs suggest older adults had a larger mixing cost, the analysis on log RTs showed a smaller mixing cost for older adults. It is likely that the untransformed RTs show a larger raw mixing cost for older adults as their overall RTs were much slower than that of younger adults. This large increase in overall response times in older adults was only observed in the non-linguistic task, potentially also because the time out was much longer in this task (ten seconds). We therefore also conducted an additional analysis with z-scored RTs to cancel out the overall age effect (and indeed, in this analysis there was no overall RT age-group difference). This analysis no longer showed a significant age-group difference in terms of mixing costs. In combination with the benefit older adults showed in terms of the accuracy mixing cost too, it appears that older adults’ responses were not affected more negatively by dual-rule compared to single-rule contexts than younger adults’ responses. Thus, overall, neither the linguistic nor the non-linguistic tasks showed disadvantages for older adults.

## Experiment 2

### Introduction

The noun-verb switching task showed switching and mixing costs, as well as asymmetries with larger mixing costs when using the dominant rule in a dual-rule condition. The presence of these effects is similar to effects previously observed in the bilingualism literature and could suggest some degree of top-down language control is applied when switching responses within one language too. Experiment 2 firstly aimed to compare these effects across within-language and between-language switching to examine whether language-control mechanisms are applied similarly when managing competition within one language versus between two languages. We hypothesised that if reactive control is used similarly, switching costs should be comparable across tasks. Furthermore, if proactive control is used similarly, mixing costs, and potentially the asymmetry with larger costs for the L1 or noun responses, should be comparable too across tasks. Finally, given that semantic control has been associated with language production^7^, we also included a measure of semantic control. This examined whether control is comparable when applied when suppressing alternate responses within a language, between languages, or between semantic relationships.

As a second aim, we examined how age affects within- and between-language control. Experiment 1 showed no clear language control differences between age groups, but previous literature has shown substantial age effects on bilingual control in terms of both reactive and proactive control mechanisms. If older adults show greater difficulty with proactive control, we expected their mixing costs to be larger than for younger adults. If these age-related changes are comparable across within- and between-language control, we expected these effects to be observed in both tasks, and for the age-group differences to be comparable. Similarly, if older adults show greater difficulty with reactive control, we expected their switching costs to be larger than those of younger adults, again also examining if age-related patterns are comparable for the within- and between-language control tasks.

### Methods

While Experiment 1 was not pre-registered, the pre-registration for Experiment 2 can be found at https://osf.io/rdfcs/.

#### Participants

Experiment 2 was completed by fifty older adults (*M*age = 67.20 years old, *SD* = 5.60, range = 60–82) and fifty younger adults (*M*age = 23.1, *SD* = 4.97, range = 18–35). Participants provided informed consent at the start of the study. Ethics approval was given by the ethics committee in the Department of Psychology, University of York. The study was conducted in accordance with the Declaration of Helsinki, including a pre-registration. Thirty-four of the older adults identified as female and 16 as male. Within the group of younger adults, 34 identified as female, 13 as male, and three chose one of the other options. Thirty-eight of the older adults and twenty-five of the younger adults reported having completed a graduate degree. An additional seven participants were tested but not included because their audio files were not uploaded or audible (N = 5) or because they did not follow the task instructions (N = 2). The sample size was determined based on power simulations. We first ran a simulation assessing power to detect the switching and mixing costs observed for the noun-verb task in Experiment 1. With 100 participants, power to detect these effects was over 95%. Given that Experiment 1 showed no age-group differences, we based the power in relation to age groups on the bilingual switching study with older and younger adults reported in^22^. Power to detect a mixing cost difference between age groups was over 80%. Power to detect a switching cost difference between age groups, based on effect sizes in^22^, was slightly lower (>60%). However, based on practical considerations regarding recruitment feasibility, we decided to accept this power with 100 participants.

All participants reported that they were native speakers of English and met the same inclusion criteria as reported in Experiment 1. We again used the same Prolific screening settings and eligibility criteria regarding no diagnosis of a neurodegenerative disease or cognitive impairment. We did not use a separate task of cognitive functioning due to the study being completed online. Participants were bilingual in Experiment 2 and spoke French (39 older adults; 34 younger adults) or German (11 older adults; 16 younger adults) as a second language. We recruited both French- and German-speakers to facilitate recruitment but did not aim to compare French and German speakers. Participants completed the LexTALE as a short measure of vocabulary in each language (cf.^52,53^). In this task, they saw letter strings and had to indicate whether they formed an existing word in the target language or not. Participants also completed a language background questionnaire, which included questions about age of acquisition, self-rated proficiency, language use, and language switching. Participants reported being unbalanced bilinguals, with significantly higher proficiency in and use of English than their L2 (see Supplementary Table  6). Most participants were living in an English-dominant country (typically the UK). Some participants also reported speaking more than two languages, but for the purpose of our study, we always counted French or German as the L2, depending on the language version of the task they completed. We initially pre-registered to exclude participants who scored below 50% correct on the LexTALE. However, given that we aimed for unbalanced bilinguals with a lower L2 proficiency, several participants scored below this threshold and we removed this exclusion criterion.

#### Design

Participants completed a noun-verb switching task and a bilingual switching task. The design of these tasks was the same as in Experiment 1. They also completed a measure of semantic control, which asked them to assess the relationship between words in terms of their size or colour in the presence or absence of distractors (within-subject variable: high or low control).

#### Materials

Pictures for the two switching tasks were chosen in a similar way as in Experiment 1, with the list of stimuli available on the OSF page. We used the same pictures in the bilingual and in the noun-verb switching task to make them more comparable. Identical cognates between the languages were avoided, which also resulted in a different set of stimuli for the noun-verb task than in Experiment 1. For some pictures, multiple verbs were possible (e.g., “chew”, “brush”, or “bite”), which we all accepted. Words were matched in terms of number of phonemes between languages or nouns/verbs, but the verb and L2 frequency was significantly higher than the noun and L1 frequencies. Materials for the semantic control task were based on^7^. Again, two cues per switching task were used. The cues for the noun-verb switching task were the same as in Experiment 1. For the bilingual switching task, we could not create two written cues per language and we therefore worked with two visual cues in the form of two versions of the country flag associated with the language.

#### Procedure

Participants were recruited through Prolific.co and through our existing database of older adult volunteers. They completed the study on Gorilla.sc. The procedure of the study was similar to Experiment 1, with the order of the noun-verb and bilingual switching tasks counterbalanced across participants. The tasks looked the same as in Experiment 1, with the change that each picture was presented for 3000 ms. After the two switching tasks, participants completed a semantic control task based on^7^. In this task, participants saw a target word and two or four answer options. They had to choose the answer option that was similar in either size (e.g., donkey–bicycle) or colour (e.g., cloud-tooth). Other answer options were either neutral (congruent trials) or distractors (incongruent trials) that were related to the target in another way. For example, on the incongruent trial asking what is most similar to “gym” in size, participants had to choose “theatre” while ignoring the related word “basketball”. Half of the trials were incongruent while the other half were congruent. There was no time limit on this task. After completing these tasks, participants completed the LexTALE tasks as a measure of vocabulary and the language background questionnaire, as described in the Participants section.

#### Data analysis

Data analyses were similar to Experiment 1. We first examined age effects for the within-language and between-language switching tasks separately. Age effects on the RTs from the semantic control task were also analysed, with the variables age group and control condition (congruent = $-$ 0.5; incongruent = 0.5). To examine how language-control measures relate to each other and to semantic control, we also conducted four correlational analyses on within-language switching costs versus between-language switching costs; within-language mixing costs versus between-language mixing costs; semantic cost (RTs on incongruent trials minus congruent trials) versus within-language switching costs; and semantic cost versus between-language switching costs. Finally, we conducted two analyses assessing age effects across tasks. The first examined the switching costs from the bilingual and noun-verb switching task and the semantic control cost. This included the variables age group, task, and control condition (switch trials and incongruent semantic trials grouped as high control). Task was helmert coded to compare the bilingual and noun-verb switching costs to each other and the switching costs to the semantic control cost. The final analysis compared the mixing costs from the bilingual and noun-verb tasks, including age group, task (noun-verb = $-$ 0.5; bilingual = 0.5), and trial type.

### Results

#### Bilingual switching task

##### Switching analysis

***Accuracy***

Mean accuracy per condition and language group is provided in Supplementary Table  S7. The full results from the analysis are shown in Supplementary Table  S8. The bilingual switching task showed a significant switching cost in terms of accuracy, with participants making more mistakes on switch than on non-switch trials. They were also more accurate in their L1 than in their L2. Accuracy did not show any effects of, or interactions with, age group.

***Reaction times***

The full results of the switching-effect analysis can be found in Table 4, with Table 5 showing the means per condition. Participants showed a significant switching cost, with slower responses on switch trials (*M* = 1334, *SD* = 200) than on non-switch trials (*M* = 1280, *SD* = 191). Older adults (*M* = 1376, *SD* = 190) responded more slowly than the younger adults (*M* = 1236, *SD* = 169). Of main interest for the current study, older adults had a slightly higher switching cost than younger adults, but this did not reach significance (see Table 4 and Fig. 1). None of the other main effects or interactions were significant either.

**Table 4:** Outcome of the linear mixed effect models for the bilingual switching task in Experiment 2.

Fixed effects	Estimate	Standard error	t-value	*p* value
Switching analysis				
Intercept	7.142	0.016	449.407	< 0.001
Switching	0.038	0.005	7.877	< 0.001
Language	− 0.015	0.016	− 0.941	0.352
Age group	0.115	0.028	4.185	< 0.001
Switching × Age group	0.016	0.008	1.924	0.066
Switching × Language	0.01	0.013	0.83	0.415
Age group × Language	− 0.005	0.024	− 0.207	0.837
Switching × Age group × Language	0.005	0.017	0.278	0.783
Mixing analysis				
Intercept	7.04	0.017	416.586	< 0.001
Mixing	0.165	0.01	16.942	< 0.001
Language	0.038	0.016	2.356	0.021
Age group	0.085	0.027	3.18	0.002
Mixing × Age group	0.047	0.018	2.647	0.01
Mixing × Language	− 0.113	0.015	− 7.711	< 0.001
Age group × Language	− 0.014	0.03	− 0.459	0.647
Mixing × Age group × Language	0.014	0.024	0.596	0.554

The final switching model included all by-subject and by-item random intercepts and slopes, after removal of correlations. The final mixing model included all by-subject and by-item random intercepts and slopes, after removal of correlations.

**Table 5:** Mean RTs (and standard deviations) in the bilingual switching task in Experiment 2.

	Younger adults	Older adults
Single-rule		
L1	1010 (178)	1078 (165)
L2	1126 (196)	1174 (214)
Non-switch		
L1	1221 (175)	1359 (194)
L2	1212 (202)	1337 (234)
Switch		
L1	1258 (200)	1412 (188)
L2	1259 (198)	1404 (213)
Mixing cost		
L1	212 (142)	281 (152)
L2	86 (107)	162 (131)
Switching cost		
L1	37 (110)	53 (80)
L2	47 (79)	67 (89)

**Figure 2:**
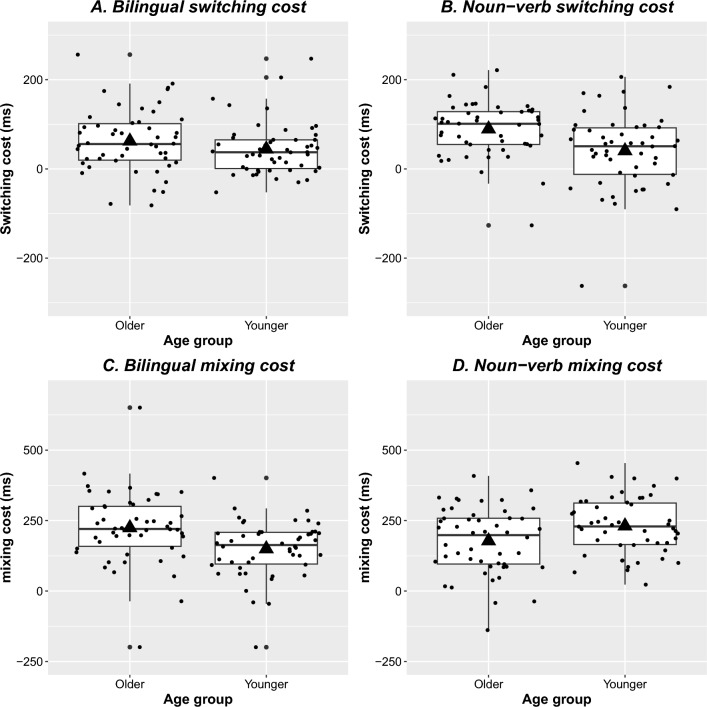
Switching costs for the bilingual (**A**, top left) and noun-verb (**B**, top right) task per age group, as well as bilingual mixing (**C**, bottom left) and noun-verb mixing (**D**, bottom right) costs. The horizontal line in the box plot reflects the median while the centre of the triangle is the mean. Each dot reflects an individual participant (jittered).

##### Mixing analysis

***Accuracy***

The full details are provided in Supplementary Tables  S7 and  S8. Participants showed a significant mixing cost, with higher accuracy on single-language trials than on dual-language trials. They were also more accurate in their L1 than L2. The mixing cost itself was also larger for the L1. The difference between languages (L1 naming advantage) was more pronounced on single-language than dual-language trials. Finally, this asymmetry in mixing cost interacted with age, as the difference between languages was largest for the younger adults.

***Reaction times***

Participants showed a significant RT mixing cost, with slower responses on non-switch trials (*M* = 1280, *SD* = 191) than on single-language trials (*M* = 1093, *SD* = 160). Across the single-language and non-switch trials, older adults (M = 1229, SD = 167) also responded more slowly than younger adults (M = 1136, SD = 145). Of main interest for the current study, mixing costs were larger in the older adults than in the younger adults (see Table 5 & Fig. 1).

Participants furthermore responded faster in their L1 (*M* = 1161, *SD* = 168) than in their L2 (*M* = 1210, *SD* = 204). The mixing cost, however, interacted with language. The mixing cost was larger for the L1 (*M*mixing cost = 246, *SD* = 151) than in the L2 (*M*mixing cost = 124, *SD* = 125). As shown in Table 5, participants were faster in their L1 than L2 in the single-language block, but not in the dual-language blocks.

Given that older adults also responded significantly slower overall than younger adults, we z-scored the RTs to account for overall slowing. These analyses aimed to make sure that the larger mixing costs (and to some extent larger switching costs) for older adults were not the consequence of their overall slower naming. These analyses still showed a significant interaction between mixing costs and age ($\beta$ = 0.225, *SE* = 0.071, *t* = 3.165, *p* = 0.002), and the interaction between switching cost and age was now also significant ($\beta$ = 0.108, *SE* = 0.038, *t* = 2.827, *p* = 0.01).

#### Noun-verb switching task

##### Switching analysis

***Accuracy***

Mean accuracy by condition is shown in Supplementary Table  S9, with full results from the analysis shown in Supplementary Table  S10. There was a switch cost in terms of accuracy, with better performance on non-switch than switch trials. This interacted with rule, such that the switching cost was larger for the nouns. There were no effects of, or interactions with, age.

***Reaction times***

The full results of the switching-effect analysis can be found in Tables 6 and 7. Participants showed a significant switching cost, with slower responses on switch trials (*M* = 1451, *SD* = 199) than on non-switch trials (*M* = 1386, *SD* = 212). Overall RTs did not differ between age groups but the switching cost was larger for older than younger adults (see Table 7 and Fig. 1). None of the other main effects or interactions were significant.

**Table 6:** Outcome of the linear mixed effect models for the noun-verb switching task in Experiment 2.

Fixed effects	Estimate	Standard error	t-value	*p* value
Switching analysis				
Intercept	7.224	0.017	429.461	< 0.001
Switching	0.048	0.007	7.363	< 0.001
Rule	− 0.003	0.019	− 0.171	0.866
Age group	0.002	0.029	0.074	0.941
Switching × Age group	0.034	0.01	3.292	0.003
Switching × Rule	− 0.031	0.016	− 2.014	0.057
Age group × Rule	0.024	0.015	1.573	0.12
Switching × Age group × Rule	0.002	0.017	0.1	0.92
Mixing analysis				
Intercept	7.117	0.018	389.847	< 0.001
Mixing	0.168	0.009	17.864	< 0.001
Rule	0.056	0.021	2.722	0.012
Age group	0.007	0.03	0.222	0.825
Mixing × Age group	− 0.044	0.017	− 2.613	0.011
Mixing × Rule	− 0.09	0.018	− 4.875	< 0.001
Age group × Rule	0.011	0.018	0.637	0.526
Mixing × Age group × Rule	0.022	0.027	0.825	0.412

The final switching model included all by-subject and by-item random intercepts and slopes, apart from the item slope for switching × age × rule. The final mixing model included all intercepts and slopes after removal of correlations.

**Table 7:** Mean RTs (and standard deviations) in the noun-verb switching task in Experiment 2.

	Younger adults	Older adults
Single-rule		
Noun	1114 (224)	1135 (200)
Verb	1232 (231)	1250 (199)
Non-switch		
Noun	1405 (228)	1355 (198)
Verb	1400 (240)	1385 (215)
Switch		
Noun	1463 (197)	1462 (199)
Verb	1421 (216)	1456 (220)
Mixing cost		
Noun	291 (151)	219 (162)
Verb	168 (126)	135 (118)
Switching cost		
Noun	58 (110)	107 (84)
Verb	21 (94)	71 (96)

##### Mixing analysis

***Accuracy***

Supplementary Tables  S9 and  S10 provide detail about the accuracy analysis. Accuracy was higher for single-rule than for non-switch trials. This mixing cost was furthermore larger for younger than older adults. Overall accuracy was higher for noun responses.

***Reaction times***

Participants showed a significant RT mixing cost, with slower responses on non-switch trials (*M* = 1386, *SD* = 212) than on single-rule trials (*M* = 1182, *SD* = 199). Of main interest for the current study, and contrary to the hypothesis, mixing costs were smaller in the older adults than in the younger adults (see Table 7 and Fig. 1).

Participants furthermore responded faster with nouns (*M* = 1245, *SD* = 196) than with verbs (*M* = 1313, *SD* = 210). The mixing cost, however, interacted with rule and was larger for nouns (*M*mixing cost = 255, *SD* = 160) than verbs (*M*mixing cost = 151, *SD* = 123). As shown in Table 7, the RT difference between nouns and verbs was reduced in the non-switch trials compared to the single-rule trials.

#### Comparisons across tasks

We also aimed to compare RTs across tasks, including the semantic control task. This task showed a main effect of age ($\beta$ = 0.293, *SE* = 0.038, *t* = 7.66, *p* <0.001), with older adults responding more slowly (*M* = 3798, *SD* = 907) than younger adults (*M* = 2836, *SD* = 551). There was also a main effect of control ($\beta$ = 0.289, *SE* = 0.053, *t* = 5.42, *p* <0.001), showing that people responded more slowly in the high-control condition (*M* = 3906, *SD* = 1151) than in the low-control condition (*M* = 2881, *SD* = 788). The control cost was numerically higher for older (*M*cost = 1360, *SD* = 937) than younger adults (*M*cost = 697, *SD* = 512), but this was not significant ($\beta$ = 0.063, *SE* = 0.037, *t* = 1.691, *p* = 0.0945)

We then examined whether RT costs across the different tasks were significantly related to each other. None of them were (bilingual & noun-verb switching costs: *r*(98) = $-$ 0.177, *p* = 0.077; bilingual & noun-verb mixing costs: *r*(98) = $-$ 0.04, *p* = 0.69; bilingual switching costs & semantic control cost: *r*(97) = 0.158, *p* = 0.119; noun-verb switching costs & semantic control cost: *r*(97) = 0.097, *p* = 0.341).

Next, the first analysis compared the switching costs and semantic control costs across tasks (see Supplementary Table  S11 for the full results). There was a main effect of task, with responses being faster in the bilingual than in the noun-verb switching task (see Tables 5 and 7) and in the switching tasks than in the semantic task, with the latter not having a time limit per trial. Semantic control costs were larger than switching costs, with no significant difference between bilingual and noun-verb switching costs. Task also interacted with age group, reflecting that the overall RT difference between younger and older adults was larger on the bilingual task than on the noun-verb switching task, and also larger on the semantic task than on the switching tasks. Overall, in line with the individual task analyses, older adults showed larger switching/semantic costs than younger adults. Importantly, interactions between age group, task, and control costs were not significant, suggesting the age-group differences in terms of switching/control costs were not significantly more pronounced in one of the tasks than in the other.

The second analysis aimed to examine whether age effects on mixing costs differed between the noun-verb and bilingual task. There was a significant mixing cost across tasks ($\beta$ = 0.168, *SE* = 0.007, *t* = 23.107, *p* <0.001), as also shown in the individual task analyses. There was also an effect of task ($\beta$ = $-$ 0.078, *SE* = 0.012, *t* = $-$ 6.555, *p* <0.001), with responses being slower in the noun-verb than in the bilingual task (see Tables 5 and 7). This interacted with age group ($\beta$ = 0.078, *SE* = 0.019, *t* = 4.024, *p* <0.001), confirming older adults were only slower than younger adults on the bilingual, but not on the noun-verb task. Finally, and of main interest, the three-way interaction between mixing, age, and task ($\beta$ = 0.094, *SE* = 0.025, *t* = 3.679, *p* <0.001) confirmed that the age effects differed between tasks, with older adults showing larger bilingual mixing costs but smaller noun-verb mixing costs (see Fig. 1) than younger adults. None of the other main effects or interactions were significant (*p*s> 0.08).

### Discussion

Experiment 2 showed language-control costs in both the noun-verb and the bilingual task. In both tasks, we observed mixing costs, switching costs, and an asymmetry in mixing costs with larger RT costs for using the more proficient (L1) or dominant (noun) response. While the L1 was much faster than the L2 in the single-language condition, there was no difference between languages in the dual-language condition (and if anything, the L2 was actually slightly faster than the L1). This was driven by L1 performance slowing down more in the dual- than single-language condition than L2 RTs. Similarly, while noun responses were much faster in the single-rule condition, the difference with verb responses was smaller in the dual-rule condition. This suggests that in both types of tasks language control was applied more strongly over one of the languages or rules, in a proactive manner as indicated by this asymmetry occurring in terms of mixing but not switching costs. These asymmetries in mixing costs, sometimes leading to reversed language dominance effects in dual-language contexts, are frequently discussed in light of inhibition (e.g.,^16^). Participants might have over-inhibited the L1 or noun responses in the dual-language/-rule condition to allow for easier use of the L2 or verb responses. Alternatively, or additionally, bilinguals might have activated the L2 or verbs more strongly in the dual-language/-rule condition to allow for easier use of both languages or rules, leading to smaller mixing costs for the L2/verbs.

We also showed age effects in both bilingual and in noun-verb switching tasks. In the noun-verb task, both mixing and switching costs were significantly affected but in different directions, with smaller mixing costs and larger switching costs for older adults. No overall age-group differences were observed in terms of general naming times across conditions. In the bilingual task, the age-group difference was as expected, with a larger mixing cost for older adults. Given that older adults were slower than younger adults overall, we also conducted analyses on z-scored RTs correcting for this overall slowing. These analyses too showed significantly larger mixing, as well as switching, costs for older adults. Older adults thus showed poorer reactive control on both tasks while only the bilingual (and not the within-language noun-verb) task showed poorer proactive control for older adults. These results will be discussed further in the General Discussion.

## General discussion

Across two Experiments, we examined age-group differences in tasks requiring participants to switch words between or within languages. Indications of language control (switching and mixing costs, as well as asymmetries with larger costs for the dominant rule) were observed in both tasks. However, these measures did not correlate between the within- and between-language tasks. Both tasks also showed age-group differences, but different patterns. For the noun-verb switching tasks, these age effects were only observed in Experiment 2 but concerned both mixing and switching, with the surprising finding that mixing costs were smaller for older adults in terms of accuracy as well as RTs. For the bilingual switching task, in contrast, larger RT mixing costs (and to some extent switching costs) were found for older adults.

### Bilingual language switching and mixing

In line with previous literature assessing bilingual language switching in older adults (e.g.,^20–22^), bilingual language control differed between age groups. This concerned the mixing costs in particular. This suggests older adults had greater difficulty monitoring cues and selecting languages accordingly in a dual-language environment. While overall mixing costs were larger for older adults, the asymmetry as such did not differ between age groups. This could suggest that older and younger adults proactively over-inhibited their L1 or over-activated their L2 in similar ways. Thus, overall control might diminish with age, but the way it is applied to each individual language might not change.

Switching costs appeared somewhat less affected by age, contrary to previous studies suggesting switching costs are most affected (e.g.,^21^ but cf.^42^). However, numerically switching costs were larger for older than younger adults too, and this reached significance in the z-scored RT analysis, suggesting that reactive control was affected in older adults too. Still, the age-group differences were most pronounced in terms of mixing costs. This is in line with task-switching literature (e.g.,^42^) suggesting that older adults experience difficulties most strongly when it comes to keeping multiple rules and goals in mind in a dual-rule context.

Asymmetries in switching or mixing costs between the languages have specifically been linked to unbalanced bilinguals who are more proficient in one language than the other. Balanced bilinguals with more comparable proficiency and use of two languages would not be predicted to show an asymmetry (as similar levels of control would need to be applied over both languages) and indeed often have shown symmetrical switching costs (e.g.,^22^). Thus, the asymmetries observed here are likely to be specific to unbalanced bilinguals. However, previous research with more balanced bilinguals (cf.^22^) has also shown larger bilingual switching and mixing costs for older than younger adults. This suggests the observed age-group differences could apply to both balanced and unbalanced bilinguals, although future research is necessary to assess a potential relationship between a bilingual’s daily-life language experiences and any age-group differences in terms of language control.

### Within-language switching and mixing

While Experiment 1 showed no age-group difference on mixing or switching costs in the within-language noun-verb switching task, Experiment 2 did. In line with the hypothesised direction, and in line with the bilingual task, switching costs were larger for older than younger adults. This suggests older adults experienced more difficulty with the reactive control needed to implement a response switch. In contrast, the mixing costs (surprisingly) were smaller for older adults, both for RTs and accuracy. In combination with the absence of age effects in Experiment 1, it is difficult to interpret why these mixing costs might be smaller. The smaller mixing costs seem a combination of older adults responding a bit slower than younger adults in the single-rule context but a bit faster in the dual-rule context. The combination of slower responses in the baseline and faster responses in the higher-control context leads to smaller mixing costs. It is possible, given the smaller cost in terms of RT and accuracy, that older adults applied more effort in the dual-rule context than younger adults, although this would raise the question why they only did this in the noun-verb but not in the bilingual switching task.

It is important to emphasise, however, that Experiment 1 did not show age effects. There are various differences between the Experiments that could perhaps explain differences in terms of results observed, although all are speculative. Although the task design was the same, we used slightly different stimuli that might have differed in difficulty level, with accuracy being slightly lower in Experiment 2. Furthermore, it is possible that monolinguals (Experiment 1) and bilinguals (Experiment 2) differ in how they use their language control or in their language processing or production more generally. Bilinguals are sometimes found to be slower in language production tasks than monolinguals (e.g.,^54^), which is line with the slower naming observed in Experiment 2 compared to Experiment 1. Bilinguals in Experiment 2 also completed the noun-verb and bilingual switching tasks in the same session, using the same stimuli. However, in the absence of correlations between bilingual and noun-verb switching, the interpretation that the noun-verb pattern differences might be due to differences between bilinguals and monolinguals appears less likely. The surprising age-group differences observed for the noun-verb switching task, in combination with the expected patterns in the bilingual switching task, thus require future research to examine if—and potentially when—older adults experience less difficulty maintaining and using two within-language rules in mind than younger adults.

### Within- versus between-language switching

The within- and between-language switching tasks captured the same type of basic costs, including switching and mixing costs and an asymmetry in the mixing costs. Age effects were observed in both tasks and were comparable in terms of switching costs, which were larger for older than younger adults in both studies. Furthermore, the comparison between tasks showed that the age effect on switching cost did not differ across experiments. This suggests that older adults needed more time to implement reactive control both when switching within a language and when switching between languages.

However, the switching costs did not correlate significantly across tasks, suggesting the underlying reactive control mechanisms might be different. Furthermore, while older adults showed larger bilingual mixing costs, they showed smaller mixing costs than younger adults in Experiment 2’s noun-verb task. Together, these patterns suggest that language control as applied during within- versus between-language switching is different, at least in the type of task we assessed. This questions whether language control relies fully on domain-general mechanisms and instead suggests it might be applied in a task-specific manner. Furthermore, the type of control needed to manage competition between semantic relationships might also differ from the control used when switching between response types or languages. Although previous research^7^ has shown relationships between semantic control and coherency in language production, our observed switching costs did not correlate with semantic control.

Nevertheless, future research is needed to examine different types of within-language competition. In the current study, we chose noun-verb switching to ensure we had one dominant rule (nouns), similar to the unbalanced bilinguals having one dominant language. However, in daily-life, nouns and verbs are not necessarily in competition with each other. For instance, words like “climb” and “ladder” will often be used together within a sentence. Therefore, it is possible that the competition created within our task is not the type of within-language competition that speakers usually resolve in natural conversations.

In conclusion, the way control changes with age depends on the way language users need to manage competition between words. Within bilingual environments requiring control over competition between two languages, older adults experience greater difficulty using two languages in response to cues. However, controlling competition between words within one language might not always be negatively affected by age.

### Supplementary Information

Supplementary Information.

## Data Availability

A computationally reproducible version of this paper is available at: https://github.com/AMTdeBruin/Bilingual-switching-ageing. Stimuli, data, and analysis scripts are available at the above link and, together with the pre-registration for Experiment 2, at DOI 10.17605/OSF.IO/RDFCS.
