# Supplementary material for: A comparison of language control while switching within versus between languages in younger and older adults
Source: Sci Rep. 2023 Oct 5;13:16740. doi: 10.1038/s41598-023-43886-1 (PMC10555994; doi:10.1038/s41598-023-43886-1)
Supplement: Supplementary file 1 — Supplementary Information. [file 41598_2023_43886_MOESM1_ESM.pdf]

5 Supplementary materials

Manuscript title: A comparison of language control while switching within versus between languages in younger and older adults. Authors: Angela de Bruin, Heidi Kressel, & Daisy Hemmings

Experiment 1 Linguistic noun-verb switching task

Table S1: Mean accuracy (and standard deviations) in the noun-verb switching task in Experiment 1.

	**Younger adults**	**Older adults**
**Single-rule**		
Noun	97% (4.5)	96.3% (4.7)
Verb	95.6% (5.4)	94.6% (9.7)
**Non-switch**		
Noun	94.8% (4.6)	95.1% (3.8)
Verb	94.8% (5.4)	94.9% (10.1)
**Switch**		
Noun	92.8% (7)	94% (4.8)
Verb	93.4% (6.6)	93% (12.3)
**Mixing cost**		
Noun	-2.2% (5.1)	-1.2% (4.6)
Verb	-0.8% (6.1)	0.2% (4.3)
**Switching cost**		
Noun	-2% (6.4)	-1.2% (4.1)
Verb	-1.5% (6)	-1.8% (4.1)

Table S2: Outcome of the generalised linear mixed effect models for the switching and mixing accuracy effect of the linguistic noun-verb switching task in Experiment 1. The final switching model included by-subject and by-item random intercepts, as well as by-subject slopes for switching and rule. The final mixing model included by-subject and by-item random intercepts, as well as all by-subject slopes and by-item slopes for age group, rule, mixing x rule, and age group x rule.

**Fixed effects**	**Estimate**	**Standard Error**	**z-value**	**p-value**
Switching cost analysis				
Intercept	3.37	0.172	19.622	<0.001
Switching	-0.288	0.084	-3.443	<0.001
Rule	0.197	0.143	1.384	0.166
Age group	0.227	0.225	1.009	0.313
Switching x Age group	-0.003	0.167	-0.018	0.985
Switching x Rule	0.068	0.159	0.425	0.671
Age group x Rule	0.206	0.284	0.726	0.468
Switching x Age group x Rule	-0.266	0.317	-0.84	0.401
Mixing cost analysis				
Intercept	4.017	0.201	20.002	<0.001
Mixing	-0.33	0.102	-3.223	0.001
Rule	-0.326	0.253	-1.288	0.198
Age group	0.444	0.301	1.473	0.141
Mixing x Age group	0.296	0.202	1.465	0.143
Mixing x Rule	0.485	0.295	1.645	0.1
Age group x Rule	-0.154	0.43	-0.359	0.72
Mixing x Age group x Rule	0.095	0.427	0.223	0.823

Non-linguistic switching task

Table S3: Mean accuracy (and standard deviations) in the non-linguistic switching task in Experiment 1.

	**Younger adults**	**Older adults**
**Single-rule**		
Size	96.1% (9.3)	94.1% (11.6)
Parity	95.9% (6.4)	93.4% (8.8)
**Non-switch**		
Size	93.6% (5.3)	92.8% (12.8)
Parity	88.9% (10.8)	91.9% (9.4)
**Switch**		
Size	95.8% (3.7)	94.4% (12.4)
Parity	89.4% (9.7)	92.2% (8.3)
**Mixing cost**		
Size	-2.4% (10.7)	-1.3% (12.9)
Parity	-7.1% (8.9)	-1.5% (11.2)
**Switching cost**		
Size	2.2% (4.6)	1.5% (4.3)
Parity	0.5% (5.8)	0.3% (3.6)

Table S4: Outcome of the generalised linear mixed effect models on accuracy switching and mixing costs in the non-linguistic switching task in Experiment 1. The final switching model included by-subject and by-item random intercepts plus the participant slope for rule. The final mixing model included by-subject and by-item random intercepts and all slopes apart from the by-item slope for mixing x age x rule.

**Fixed effects**	**Estimate**	**Standard Error**	**z-value**	**p-value**
Switching cost analysis				
Intercept	3.048	0.216	14.087	<0.001
Switching	0.21	0.074	2.851	0.004
Rule	-0.603	0.1	-6.045	<0.001
Age group	0.293	0.244	1.203	0.229
Switching x Age group	-0.08	0.147	-0.546	0.585
Switching x Rule	-0.41	0.148	-2.776	0.006
Age group x Rule	0.453	0.199	2.271	0.023
Switching x Age group x Rule	0.132	0.294	0.447	0.655
Mixing cost analysis				
Intercept	3.54	0.188	18.881	<0.001
Mixing	-0.776	0.288	-2.69	0.007
Rule	-0.325	0.286	-1.138	0.255
Age group	-0.033	0.254	-0.128	0.898
Mixing x Age group	0.728	0.342	2.125	0.034
Mixing x Rule	-0.114	0.48	-0.237	0.813
Age group x Rule	0.149	0.312	0.478	0.633
Mixing x Age group x Rule	0.749	0.507	1.479	0.139

Table S5: Outcome of the linear mixed effect models on RT switching and mixing costs for the non-linguistic switching task in Experiment 1. The final switching model included by-subject and by-item random intercepts and slopes, apart from the by-item slope for age x switching x rule. The final mixing model included by-subject and by-item intercepts and all slopes apart from the by-item slopes for rule x age and mixing x age.

Fixed effects	Estimate	Standard Error	t-value	p-value
Switching cost analysis				
Intercept	7.355	0.022	327.449	<0.001
Switching	-0.011	0.014	-0.751	0.468
Rule	-0.001	0.041	-0.013	0.99
Age group	0.319	0.04	7.914	<0.001
Switching x Age group	0.001	0.018	0.037	0.971
Switching x Rule	0.018	0.019	0.958	0.361
Age group x Rule	0.033	0.023	1.433	0.164
Switching x Age group x Rule	0.031	0.024	1.282	0.204
Mixing cost analysis				
Intercept	7.193	0.023	307.937	<0.001
Mixing	0.334	0.017	19.769	<0.001
Rule	-0.015	0.044	-0.353	0.734
Age group	0.359	0.042	8.485	<0.001
Mixing x Age group	-0.084	0.028	-2.999	0.004
Mixing x Rule	0.014	0.018	0.765	0.452
Age group x Rule	0.017	0.018	0.958	0.341
Mixing x Age group x Rule	0.006	0.033	0.171	0.865

Experiment 2

Table S6: Summary of the participants' language proficiency in their L1 (English) and L2 (French or German). Means (and standard deviations) are provided for the LexTale measure of vocabulary, self-rated proficiency, language use during childhood (based on four questions on childhood and teenagers years at home and at school), language use during adulthood (based on 16 questions including different contexts, interlocutors, and activities), and daily-life switching in general, within a conversation, and within a sentence. Age groups did not differ significantly on any of the use or switching ratings. All proficiency measures showed significantly higher L1 than L2 proficiency. Older adults also performed better across both languages in the LexTale, but this did not interact with language. Self-rated speaking, understanding/listening, and reading did not differ between the age groups. Self-rated writing was lower for older adults, but given that our tasks did not use or assess writing, this was not problematic for the current study.

	**Younger adults**	**Older adults**
**L1: self-rated proficiency (1-10)**		
Speaking	9.96 (0.2)	10 (0)
Understanding	9.96 (0.28)	10 (0)
Writing	9.88 (0.44)	9.98 (0.14)
Reading	9.94 (0.42)	10 (0)
**L2: self-rated proficiency (1-10)**		
Speaking	5.96 (2.03)	6.34 (2.02)
Understanding	7.12 (1.72)	7.02 (1.97)
Writing	6.5 (1.93)	5.52 (2.14)
Reading	7.46 (1.86)	7.3 (1.98)
**Language use (1=all French/German, 5=all English)**		
Childhood	4.32 (0.61)	4.48 (0.56)
Adulthood	4.51 (0.35)	4.46 (0.53)
**LexTale (0-100%)**		
English	89.03 (9.95)	95.72 (6.29)
French/German	59.73 (11.06)	63.85 (13.44)
**Daily-life language switching (1=never, 7=very frequently)**		
Daily	2.74 (1.52)	2.52 (1.54)
Within a conversation	3.36 (1.86)	3.32 (1.89)
Within a sentence	2.76 (1.74)	2.64 (1.79)

Bilingual switching task

Table S7: Mean accuracy (and standard deviations) in the bilingual switching task in Experiment 2.

	**Younger adults**	**Older adults**
**Single-language**		
L1	98.7% (2.5)	96.6% (6)
L2	92.5% (7.7)	89.4% (12.1)
**Non-switch**		
L1	95.7% (6.2)	94.6% (8)
L2	93.2% (8.6)	88.9% (12.8)
**Switch**		
L1	94.9% (6.6)	93.1% (8.3)
L2	92.2% (8.7)	88.3% (12.8)
**Mixing cost**		
L1	-3% (5.3)	-2% (5.2)
L2	0.7% (4.9)	-0.5% (6.9)
**Switching cost**		
L1	-0.8% (5)	-1.6% (4.1)
L2	-1% (4.8)	-0.7% (5.8)

Table S8: Outcome of the generalised linear mixed effect models for the switching and mixing accuracy effect of the bilingual switching task in Experiment 2. The final switching model included by-subject and by-item random intercepts, and the by-subject slope for language. The final mixing model included by-subject and by-item random intercepts, all by-subject slopes and by-item slopes for age, language, mixing x age, and age x language.

Fixed effects	Estimate	Standard Error	z-value	p-value
Switching cost analysis				
Intercept	3.313	0.171	19.368	<0.001
Switching	-0.179	0.067	-2.691	0.007
Language	-0.53	0.142	-3.721	<0.001
Age group	-0.301	0.248	-1.216	0.224
Switching x Age group	-0.011	0.133	-0.082	0.935
Switching x Language	0.167	0.133	1.254	0.21
Age group x Language	-0.257	0.284	-0.903	0.366
Switching x Age group x Language	0.195	0.266	0.733	0.464
Mixing cost analysis				
Intercept	3.783	0.182	20.79	<0.001
Mixing	-0.44	0.087	-5.083	<0.001
Language	-1.063	0.258	-4.125	<0.001
Age group	-0.391	0.271	-1.445	0.149
Mixing x Age group	0.27	0.17	1.586	0.113
Mixing x Language	0.969	0.18	5.372	<0.001
Age group x Language	0.088	0.36	0.243	0.808
Mixing x Age group x Language	-0.943	0.343	-2.751	0.006

Noun-verb switching task

Table S9: Mean accuracy (and standard deviations) in the noun-verb switching task in Experiment 2.

	Younger adults	Older adults
**Single-rule**		
Noun	96.4% (7.1)	95.3% (10.1)
Verb	93.6% (7.7)	94.3% (7.7)
**Non-switch**		
Noun	93.6% (7.1)	95.3% (7.1)
Verb	91.7% (8.4)	93.4% (6.5)
**Switch**		
Noun	91% (8.3)	91.4% (8.8)
Verb	89.4% (9.6)	92.3% (7.2)
**Mixing cost**		
Noun	-2.8% (8.5)	-0.1% (9)
Verb	-1.8% (5.9)	-0.8% (6.3)
**Switching cost**		
Noun	-2.6% (7.5)	-3.9% (5.2)
Verb	-2.3% (7.2)	-1.2% (5.5)

Table S10: Outcome of the generalised linear mixed effect models for the switching and mixing accuracy effect of the linguistic noun-verb switching task in Experiment 2. The final switching model included by-subject and by-item random intercepts, all by-subject slopes, and by-item slopes for switching, rule, age group, and age group x rule. The final mixing model included by-subject and by-item intercepts, all by-subject slopes, and by-item slopes for mixing, age group, rule, and mixing x age group x rule.

**Fixed effects**	**Estimate**	**Standard Error**	**z-value**	**p-value**
Switching cost analysis				
Intercept	2.939	0.123	23.946	<0.001
Switching	-0.43	0.082	-5.26	<0.001
Rule	-0.112	0.173	-0.647	0.517
Age group	0.29	0.194	1.5	0.134
Switching x Age group	-0.105	0.143	-0.732	0.464
Switching x Rule	0.286	0.132	2.165	0.03
Age group x Rule	0.089	0.184	0.483	0.629
Switching x Age group x Rule	0.41	0.262	1.563	0.118
Mixing cost analysis				
Intercept	3.625	0.159	22.813	<0.001
Mixing	-0.472	0.121	-3.888	<0.001
Rule	-0.394	0.199	-1.978	0.048
Age group	0.178	0.247	0.721	0.471
Mixing x Age group	0.478	0.234	2.043	0.041
Mixing x Rule	0.288	0.203	1.418	0.156
Age group x Rule	0.118	0.231	0.512	0.608
Mixing x Age group x Rule	-0.464	0.504	-0.921	0.357

Semantic control task

Table S11: Outcome of the linear mixed effect model comparing the switching costs and semantic control cost across the three tasks. The final switching model included by-subject and by-item random intercepts and by-subject slopes for difficulty and task. Task was helmert coded; task 1 refers to bilingual vs noun-verb switching while task 2 refers to the switching tasks vs the semantic control task.

Fixed effects	Estimate	Standard Error	t-value	p-value
Intercept	7.486	0.024	315.600	<0.001
Control (high/low)	0.104	0.010	10.824	<0.001
Task 1: Bilingual vs Noun-Verb	0.080	0.010	8.188	<0.001
Task 2: Switching vs Semantic	0.784	0.023	33.621	<0.001
Age group	0.134	0.025	5.290	<0.001
Control x Age group	0.035	0.007	4.975	<0.001
Control x Task 1	0.010	0.006	1.743	0.081
Control x Task 2	0.175	0.028	6.278	<0.001
Age group x Task 1	-0.118	0.019	-6.064	<0.001
Age group x Task 2	0.233	0.035	6.694	<0.001
Control x Age group x Task 1	0.018	0.011	1.596	0.11
Control x Age group x Task 2	0.028	0.015	1.850	0.064
